# The Ontogeny of Bumblebee Flight Trajectories: From Naïve Explorers to Experienced Foragers

**DOI:** 10.1371/journal.pone.0078681

**Published:** 2013-11-12

**Authors:** Juliet L. Osborne, Alan Smith, Suzanne J. Clark, Don R. Reynolds, Mandy C. Barron, Ka S. Lim, Andy M. Reynolds

**Affiliations:** 1 Environment & Sustainability Institute, University of Exeter, Penryn, Cornwall, United Kingdom; 2 Rothamsted Research, Harpenden, Hertfordshire, United Kingdom; 3 Natural Resources Institute, University of Greenwich, Chatham, Kent, United Kingdom; 4 Landcare Research, Lincoln, New Zealand; Ghent University, Belgium

## Abstract

Understanding strategies used by animals to explore their landscape is essential to predict how they exploit patchy resources, and consequently how they are likely to respond to changes in resource distribution. Social bees provide a good model for this and, whilst there are published descriptions of their behaviour on initial learning flights close to the colony, it is still unclear how bees find floral resources over hundreds of metres and how these flights become directed foraging trips. We investigated the spatial ecology of exploration by radar tracking bumblebees, and comparing the flight trajectories of bees with differing experience. The bees left the colony within a day or two of eclosion and flew in complex loops of ever-increasing size around the colony, exhibiting Lévy-flight characteristics constituting an optimal searching strategy. This mathematical pattern can be used to predict how animals exploring individually might exploit a patchy landscape. The bees’ groundspeed, maximum displacement from the nest and total distance travelled on a trip increased significantly with experience. More experienced bees flew direct paths, predominantly flying upwind on their outward trips although forage was available in all directions. The flights differed from those of naïve honeybees: they occurred at an earlier age, showed more complex looping, and resulted in earlier returns of pollen to the colony. In summary bumblebees learn to find home and food rapidly, though phases of orientation, learning and searching were not easily separable, suggesting some multi-tasking.

## Introduction

Constantly changing temporal and spatial distributions of resources provide complex challenges to animals. Understanding how they explore the landscape can give insight into how they find and selectively exploit these resources efficiently. The impressive abilities of bumblebees and honeybees to exploit a landscape for nectar and pollen for their colony have been investigated in terms of their ability to learn and memorize visually complex routes in pursuit of these rewards, their sophisticated spatial navigational abilities, and their energetic efficiency at reward collection [1]–[5]. However, most deductions have been made without researchers being able to map the complete flight paths taken by bees in real landscapes whilst they learn, search and forage. Instead, researchers have analyzed detailed sections of flight such as flying near the colony entrance [6]–[9] or at flower patches [5], [10], [11], or designed elegant experiments to measure flight characteristics in a simulated foraging environment [12]–[15]. The objective of this study was, for the first time, to map and characterize the flights of bumblebee workers, starting with naïve bees on their first exploratory flights. We examined whether the shapes of these flights indicate an optimal strategy for searching or learning, and analyzed the changes in flight trajectories with experience as they developed into successful foraging flights.

### Learning About the Colony Entrance

When a bumblebee first leaves the colony, she makes short flights which have been described as ‘learning flights’ or ‘orientation flights’ [6], [8], [16]. Learning flights in social and solitary Hymenoptera start with circumscribed movements close to the nest, backing away in a series of zigzags or arcs of constant angular velocity, but increasing radius, roughly centered on the entrance hole [16]. During these arcing maneuvers a bee gathers visio-spatial information (and possibly olfactory information) relating to the colony entrance and nearby landmarks to enable a successful return at the end of a trip (reviewed in [8], [9]). The description of these ‘learning flights’ has previously focused on the portion visible to an observer or video at the colony [8], [9], [17], and indeed sometimes the flights only cover this short range. However, the bee may fly beyond view and there are no published data on what the bees do next. During the ‘unseen’ portions of these preliminary flights, away from the colony entrance, not only is the bee likely to be learning the landscape, but it is also the bee’s first opportunity to search for flowers and to manipulate flowers to gather nectar and pollen. Since the flights studied here are likely to include learning, orientation, searching and possibly some foraging; then we refer to them as ‘preliminary flights’ rather than ‘orientation flights’ to avoid confusion with previous literature.

### Exploring the Landscape and Searching for Forage

How do bumblebees *explore* and *choose* where to forage in a heterogeneous environment? They show constancy to plant species and to forage area over several days [1], [4], [18], but how do they make these choices in the first place? As [19] note with respect to honeybees *“little is known about the actual process of searching, because of the difficulty of following individual bees in the field”*. Does a bumblebee, leaving the nest for the first time, fly in one direction until suitable forage is reached and then start feeding? Or does the bee make several flights to learn about the vicinity before starting to forage? In exploring, they may use an optimal strategy in terms of the energy and time utilized to find patches of flowers, such as a random walk, or spiral pattern or random looping pattern [20]–[22]. Honeybees fly in distinctive looped search patterns when attempting to locate their hive, after their hive-centred navigation mechanisms have been disrupted [23], and when attempting to relocate a food source [24] and the tendency for loop sizes to increase over time results in ‘scale-free’ (Lévy flight) characteristics. This strategy is considered optimal in these circumstances because (a) it ensures that the area where the target is expected to lie is searched most intensively [25], and (b) the bee has a low chance of getting lost by centring the search on a known location. We looked for evidence of these characteristics in bumblebees on their preliminary flights.

In contrast to honeybees, bumblebees cannot rely on nest-mates to tell them the location of suitable forage, although they do communicate olfactory information about forage in the area [26]. Characterization of these early bumblebee flights will provide some insight into how they compare with the preliminary flights of honeybees [17], [27] that can acquire spatially explicit information on forage sources from dancing nest-mates in the colony [28]. We used harmonic radar [29], [30] to plot the flight trajectories of individual bumblebee workers (*Bombus terrestris* L.) with increasing experience, from naïve bees to experienced foragers. We analysed the flight patterns for evidence of learning, searching, and the tracks were superimposed on the landscape for evidence of the start of foraging.

## Methods

A *Bombus terrestris* L. colony (supplied by Koppert BC), consisting of one queen and about 50 workers, was placed at the edge of a field on Rothamsted Research Farm (Hertfordshire, UK). A transparent Perspex® tunnel with moveable doors was mounted at the front of the colony so that bees could be removed and replaced as necessary. The bees could forage freely in the surrounding arable landscape and were given no extra food supply. Some pupae from the colony were kept in an incubator and newly hatched workers were marked on the thorax with numbered discs (Opalithplättchen, EH Thorne Ltd, Lincoln, UK) and introduced to the field colony each day of the study so there was a continual supply of naïve bees that would be taking preliminary flights. Two observers and an sVHS colour video camera recorded all departures and arrivals of marked bees to the colony from dawn to dusk (04∶00 h –21∶30 h) over eleven days (17–27 June) to document trip histories for each individual bee over the study period. These data were used to summarize the age of first flight, duration and timing of preliminary flights, the number of flights per day per bee, and the number of flights before an individual returned with pollen loads.

The harmonic radar [30], [31] was positioned 281 m away from the colony (Figure S1), and used to track individual marked bees with varying flight experience. When an individual marked bee left the colony box for the first time and entered the Perspex tunnel, she was briefly captured and a transponder (16 mm long, weighing 6–10 mg; [29]) was attached to her thorax on top of the numbered disk. The bee with transponder was then placed at the open end of the tunnel and was free to fly. Her flight was tracked with the radar, using the reflected radar signals to record a range and bearing of the bee’s location every 3 seconds. The transponders could be detected within a circle of radius ∼700 m centred on the radar, and an altitude of ∼1–6 m. The radar detects signals over this range on a ‘line of sight’ basis so that if the bee flies over a hedge or obstacle, or lands on the ground then no radar signal was detected. When the bee returned to the tunnel entrance at the end of her flight, the transponder was removed so that she could re-enter the colony un-hampered. Each bee was only tracked once, and they had a range of experience. Tracking was done in dry bright conditions.

The landscape over which the bees were tracked was a relatively flat area comprising arable crops and partially bounded by hedges (Figure S1; as used in previous studies [27], [29]). During the tracking period, the floral resources within 1 km of the colony were mapped (Figure S1). The most abundant floral resources providing nectar and pollen in the radar-visible area were plots of the crops *Vicia faba* L. (field beans) and *Lupinus albus* L. (lupins) and some flowering plants along field edges. There were gardens outside the radar visible area which contained flowering plants attractive to bees, but these could not be quantified. Evidence for foraging was considered direct if the bee was either seen foraging during its tracked flight or returned with pollen (subsequently identified by colour and morphology). Indirect evidence for foraging was defined as a gap in radar signals (over 3 minutes) mapped to a location with suitable foraging resource in the landscape.

## Analyses

Flight tracks (N = 38) were included in all analyses if the bees returned to the colony on the same day as they left, and they were radar-detectable for all or most of the flight (n = 28). If the bee did not return the same day; or was not tracked when it did return (in three cases the transponder was detached or damaged) then the tracks (10) were only used for estimates of ground speed and arcing behaviour. The tracks were split into three groups according to the experience of the bee. Group 1 comprised bees on their 1^st^ trip; Group 2 included bees on their 2^nd^ or 3^rd^ trip; Group 3 included bees that were considered experienced foragers and had flown a minimum of 6 trips before tracking (Table S1). Unfortunately, no bees on their 4^th^, 5^th^ or 6^th^ trips were tracked due to the difficulty of measuring trip number accurately in real time whilst actively tracking. For each trip we calculated the maximum displacement distance from the colony, the total distance travelled during the flight, the average groundspeed (average speed of flight between two consecutive radar signals or ‘fixes’ of the bee’s position), the area of the convex hull polygon encompassing all the fixes for that flight, and the interquartile range (IQR) of bearings from the colony (encompassing 50% of the fixes). The angular range over which the bee flew from the colony was also approximated by recording the number of quadrants in which radar fixes occurred for each track (Table S1). Kruskal-Wallis one-way analysis of variance was used to compare bees of different experience for each of these track parameters (test statistic H, approx. ∼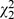) which is a robust test for small sample sizes. Since this non-parametric test is based on ranks, we have consistently reported medians (rather than means) as averages.

If tracking is constant and unobstructed, then the radar will return a fix every 3 seconds. Sometimes a sequence of missing fixes creates a gap in the track which leads to uncertainty about the bee’s location and/or activity. For example, the bee could be resting (hidden from radar view in grass or low vegetation), flying out of radar view (high/low/behind a hedge) or could be foraging undetected. If the transponder is not aligned vertically (as it is when the bee is in flight), then this can lead to a reduced signal. The gaps affect the estimation of the total distance travelled by a bee and the maximum displacement from the colony so care is needed with interpretation. We only calculated groundspeed between radar fixes where the gap was less than 15 seconds.

We used the following criteria to describe the shape of the flight. A bee was described as ‘*arcing*’ (a) when she was visually recorded as flying typical ‘Turn Back and Look’ zigzags within 2 m of the colony (sic [9], [16], [32]) and (b) when the radar track showed her flying within 10 m of the colony and seemingly pivoting around the entrance (where two or more consecutive turning angles were 135–225°). *Loops* are defined as sections of a track where the bee flies away from colony and then returned to the location of the colony but did not land.

### Detecting the Presence of Lévy Flight Characteristics

**Figure 1:**
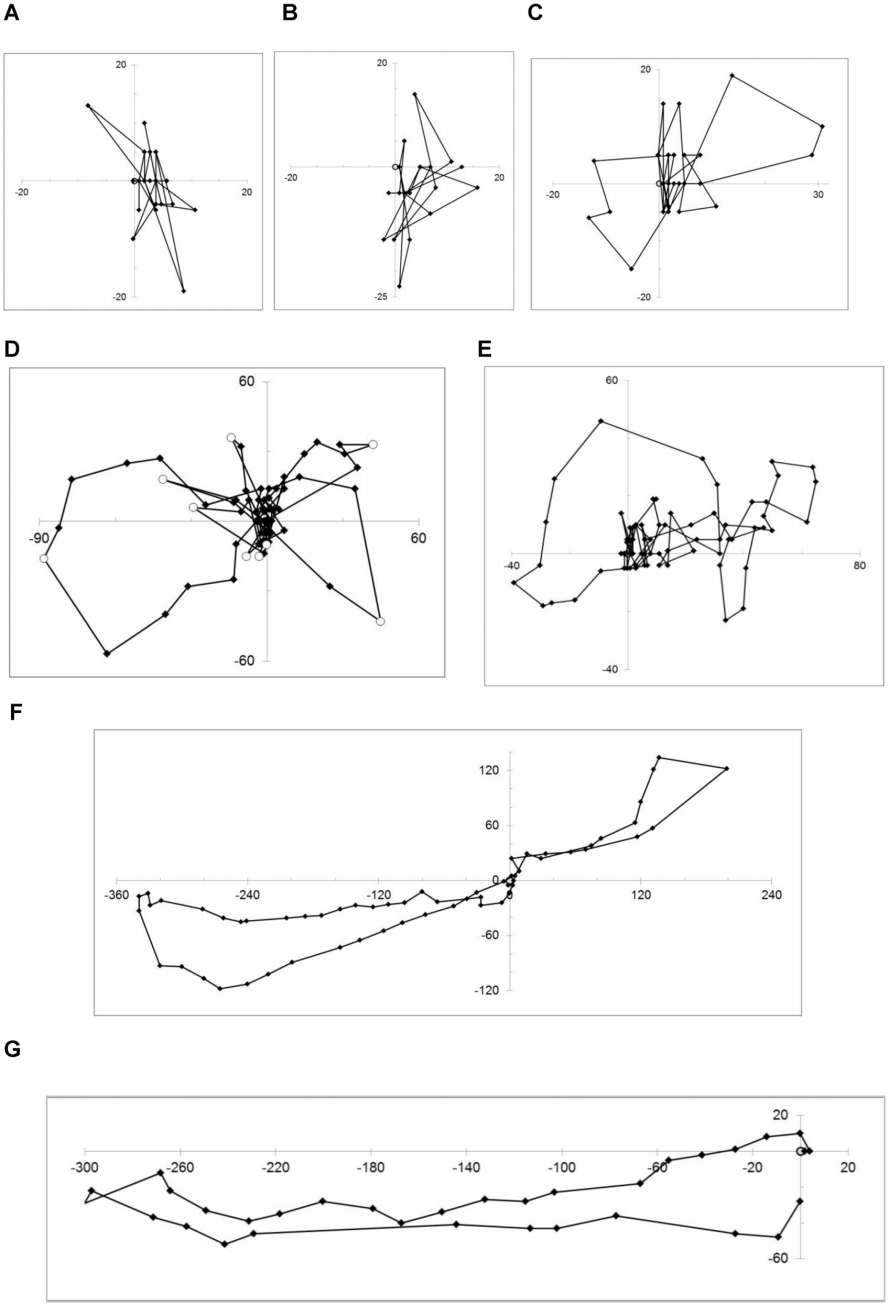
Examples of the ontogeny of bumblebee flights. The sequence and directions of each flight can be seen in detail in the video files in Figure S2. Origin is location of the colony (marked with grey filled circle). Axes show distance from colony (m). ◊ = Radar fixes, joined with black line. A – E) Flights of five individuals on their first flights; F) Bee on third flight from colony; G) Experienced forager demonstrating directed flight away from colony and directed flight in return. In Figure 1D, for the purposes of Lévy flight analyses, significant turns (○) are deemed to arise at the furthest reaches of the loops.

For the 14 complete first trips in Group 1, each of the flight trajectories was represented by a sequence of straight-line movements between points at which significant changes in direction occurred. A significant change in flight direction was deemed to have arisen at the furthest reaches of the loops (Figure 1D). Two different distributions with a power-law tail and an exponential tail were fitted to the loop length data. The power-law tail is consistent with the presence of Lévy flight patterns, an exponential tail is not. The Akaike information criterion (AIC) was used to test whether the loop-length data provide more evidence for distributions, 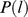, of loop-lengths 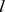, having power-law
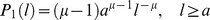



or exponential tails
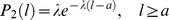
where 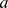 is the start of the tail, here estimated from a plot of the loop-length distribution, and 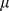 and 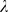 are maximum likelihood estimates (MLE) for the Lévy exponent and the exponential decay rate. The Akaike weight for a power-law tail can vary from 0 (no support) to 1 (complete support).

The goodness of fit of the data to the maximum likelihood inverse power-law distribution was tested using a Monte Carlo approach advocated by [33] which yields a *p*-value. The best-fit inverse power-law distribution is rejected if 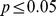, otherwise it is accepted as being plausible. The goodness of fit is illustrated by plotting the survival function (the complement of the cumulative distribution function). To construct the survival function, the simulation data 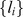 is first ranked from largest to smallest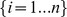. The probability that a length is greater than or equal to 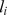 (the survival function) is then estimated as 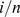.

Additionally we present the results of a ‘first significant digit’ analysis, a ‘time-series’ analysis and a spectral analysis. These analyses can cleanly distinguish between Lévy flight and strong alternative models of movement pattern data [34].

If the loop-lengths are 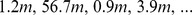
then the leading first significant digits, 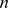, are 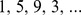 The first significant digit distribution, 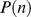, for a Lévy flight is_
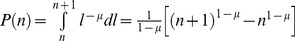
_ which is a generalization of Benford’s first digit law [34].

The time-series analysis is based on the fact that the number of turning points occurring within the time intervals t to 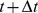 defines a time series,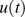, and an associated net ‘displacement’, 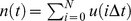. If the values of 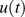 are completely uncorrelated and behave like ‘white noise’, then the root-mean-square displacement 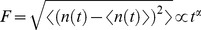 where 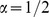 and where the angular brackets denote an ensemble average over all flights in the data set [35]. Short-term correlations in the data may cause the initial slope of a plot of 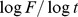 to differ from ½, although it will still approach ½ at longer times. Long-term power-law correlations that are indicative of Lévy flight characteristics will, however, generate α values ≠ ½. It can be shown that 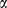 approaches the limit 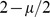 asymptotically for sufficiently long sequences and that 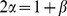 [36], [37].

The power-spectra of 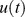 is the square of the magnitude of the Fourier transform of 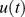:
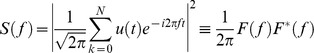
where 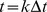 is the time at which the k^th^ positional fix was made, 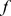 is frequency, 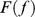 is the discrete Fourier transform of 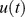, and 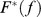
is its complex conjugate. The power-spectrum of a Lévy flight exhibits power-law scaling 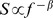 (with 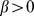), so-called ‘1/f’ noise, and is distinctly different from white noise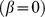 spectra that characterize flight lengths drawn from an exponential distribution or mixtures of exponential distributions [34].

## Results

### Timing of First Flights by Naïve Bees

Of 38 naïve workers added to the colony, each of which had at least 5 days in the colony over the experimental period, 28 (74%) made their first flight within 4 days of eclosion. Six made their first flights more than four days after hatching, and four such workers never left the nest during the experimental period. Of the 28 that flew in the first four days, two made their first flight within a day of hatching and 21 made their first flight 2–3 days after hatching. Of 30 bees for which there were accurate time records, the median hour (10 bees) for the first trip was between 12∶00 h and 13∶00 h (range 06∶00 h – 18∶00 h). Duration of first trips was recorded accurately for 24 bees and 83% (20 bees) lasted under 20 minutes (although showing a huge range: 10 sec–75 min). Once a bee had made her first flight, she made several trips per day and six bees (out of 30) started collecting pollen on the same day as the first flight. No records of nectar collection were made so it was not possible from colony entrance data to determine on which trip the bees started foraging. Twenty seven (out of 30) bees, brought pollen back to the colony within 10 trips. Seven of these bees started collecting pollen during trips 1–3 (one bee on first trip; six bees on third trip).

### Changes in Flight Parameters with Experience

**Figure 2:**
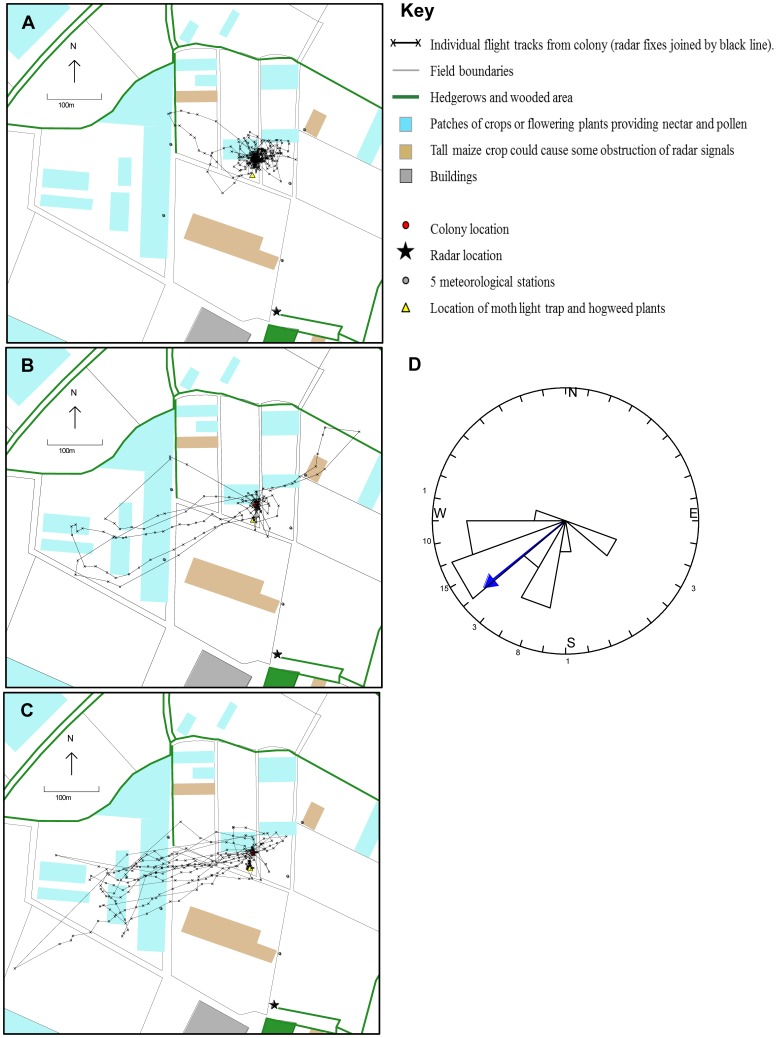
Plots of radar tracked flights (complete) overlaid on the landscape. A) Group 1: bees on first trips (n = 14); B) Group 2: bees on 2^nd^ & 3^rd^ trips (n = 6); C) Group 3: experienced bees that have flown over 6 trips (n = 8). For detail of each flight see videos in Figure S2 and maps in Figure S3. D) Circular histogram of mean wind directions (in degrees) recorded during each tracked flight, demonstrating the wind was usually from the West South West during the study (blue arrow = circular mean). Numbers on outside of circle indicate number in each bin. Means were calculated from wind direction records taken at 10 second intervals at each of five meteorological stations.

**Figure 3:**
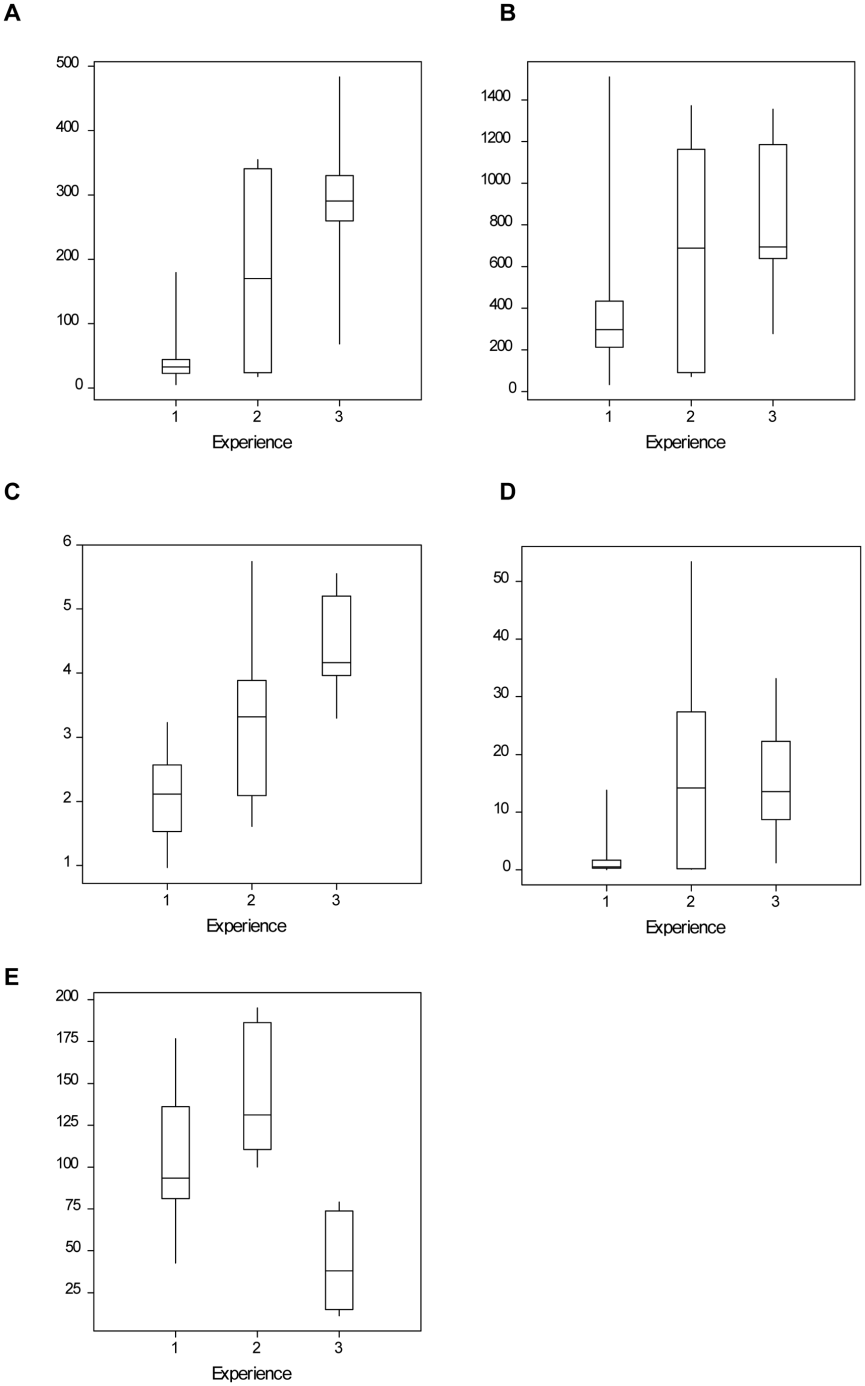
Box and whisker plots for key parameters characterizing flights of bees with differing experience. Each shows median, 25% and 75% quartiles and range (N = 28). x axis: Group 1 = 1^st^ trip, Group 2 = 2^nd^–3^rd^ trips, Group 3 = >6 trips. A) Maximum displacement from the colony (m); B) Total distance travelled during tracked trip (m); C) Average ground speed (ms^−1^) calculated for each tracked step where time gap was less than 15 s (N.B. for this graph N = 38 as incomplete tracks are included; see Table S1); D) Area (m^−2^) of convex hull polygon encompassing all radar signals for a tracked trip; E) Interquartile range (degrees) of bearings of all radar signals for a trip.

Animations of the 28 complete tracked flights (Figure S2) illustrate clear changes in flight behaviour with experience. These are summarized with the example tracks in Figure 1; and in Figure 2 which shows the extent of the use of the landscape by bees of differing experience. The maximum displacement of a bee from the nest during a trip increased significantly with the bee’s experience (H = 12.81, *p* = 0.002, N = 28; Figure 3A). First flights (n = 14) were concentrated within 50 m of the colony (median = 33 m) (Figure 1A–E; Figure 2A). Second and third flights (n = 6) stretched further but each bee still ‘explored’ in different directions (Figure 1F; Figure 2B), whilst the flights of experienced bees (n = 8) were straightened and lengthened, in this case generally to the south west of the colony (Figure 1G; Figure 2C) (Group 3 median maximum displacement = 291 m). The total distance travelled for each track also increased, but not significantly (H = 4.343, *p* = 0.114, N = 28; Figure 3B). It should be noted that for the longer tracks both of these measures of distance are likely to be underestimated because of gaps of over 3 mins in the recorded track due to the bee being obscured from view – either by an obstacle or because it was foraging or resting amongst vegetation (Figure S3). The groundspeed of the flying bees also increased significantly with experience (H = 22.59, *p* < 0.001, N = 38; Figure 3C), although it should be noted that the naïve bees might have made loops at a fine scale not resolved by the radar so their actual flight speed may be higher than the coarse ground speed measurement. The median measured groundspeed for naïve bees (first trip) was 2.1 ms^−1^ (n = 24) and for experienced bees (group 3) was twice as fast, at 4.2 ms^−1^ (n = 11).

The area covered during a flight, measured with the convex hull polygon, differed amongst the groups of bees (H = 9.349, *p* = 0.009, N = 28; Figure 3D). The area was smallest for bees on their first trip (median 518 m^2^), and increased considerably for bees even on 2^nd^ and 3^rd^ flights (median 14208 m^2^). Measurements of interquartile range of bearings from the nest for each tracked flight showed larger angular ranges for group 1 (median 93°), and group 2 (median = 131°) and a very low range for group 3 (median = 38°) illustrating the straightening out of the flight path with experience (H = 15.55, *p* < 0.001, N = 28; Figure 3E). The number of quadrants visited during an individual flight decreased from four to one with experience (Table S1).

### Shape of Flights

We explored further whether the shape of the complete tracked flights gave an indication of a searching or learning strategy. On their first flights 17 out of 18 bees were seen flying initially in arcs or fine-scale loops perpendicular to the axis of the colony entrance, within 2 m of the observer (Table 1), matching the results of Philippides et al. [9]. The animated radar tracks (Figure S2) show evidence of similar patterns continuing beyond the visual range of the observer within 10 m of colony entrance. The fine scale arcing and looping was followed by larger loops of increasing size that started and ended at the nest and were directed in different azimuthal directions. The bees flew an average of three loops during their first trip (range 0–6; Table 1). For most Group 1 bees (9/14) there was some evidence of further small scale arcing behaviour when the bee returned to the colony entrance at the end of the flight. Bees on their second and third trips flew in fewer loops, over longer distances, with some evidence of fine scale arcing/looping behaviour at the beginning and end of the flights (Table 1; [7]). There was no evidence of the bees performing spiral search patterns as they investigated the landscape on any of the flights tracked. Experienced bees in group 3 showed virtually no arcing behaviour (Table 1). They left the colony on a direct flight and returned straight into the entrance (Figure S3).

**Table 1:** Summary of the number of tracked flights in each category of experience that display ‘Turn Back and Look’ (TBL) behaviour or arcing.

Experience group	Trip no	Visual: TBL/arcs (n)	Radar: arcs @start (n)	Radar: arcs @end (n)	Arcs @Both (n)	No. loops Median (range)
1	1^st^	17 (18)	16 (18)	9 (14)	9 (14)	3 (0–6)
2	2^nd^ & 3^rd^	5 (9)	4 (9)	2 (6)	1 (6)	2 (1–4)
3	> 6^th^	1 (11)	0 (11)	0 (8)	0 (8)	1 (1–3)

Visual = arcs recorded by observer at start of flight. Radar = arcs recorded at the start and end of the flight from the track. Number of tracks (n) vary because arcing at the beginning was identified in all tracks (N = 38) and arcing at the end for just complete tracks (N = 28).

### Preliminary Flights have Lévy Flight Characteristics

**Figure 4:**
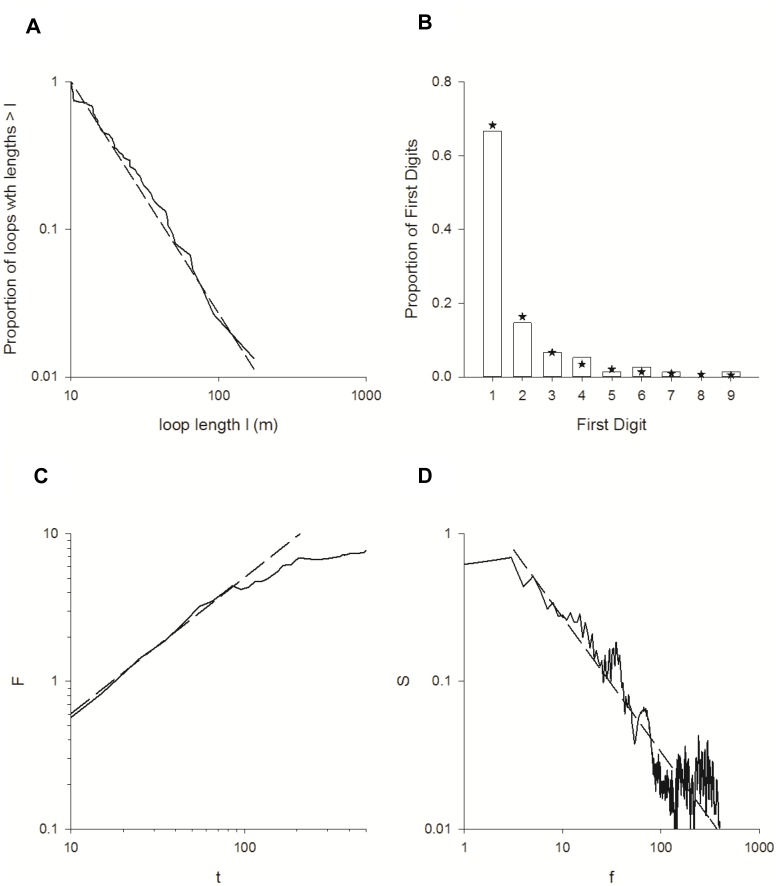
Analysis of tracks for Levy flight characteristics. A) Proportion of loops with lengths 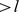
(solid-line) together with the fitted inverse power-law distribution (dashed-line). The length of the loop is the straight-line distance from the nest to a significant turn (see Figure 1D). A straight-line on the log-log plot is indicative of an inverse power-law. Here inverse power-law is evident over lengths ranging about 10 and about 120 m. B) First significant digit distributions for the observed loop lengths (histograms) and the fitted inverse power-law distribution (star) c) The net root mean square value of the running sum, 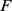 and the power spectrum of 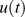. 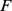 is plotted as a function of elapsed time 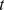, measured from the moment at which the radar first detected each released bee. The dashed line with 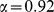 constitutes a linear least squares fit to the data (correlation coefficient, 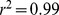) . A straight-line on the log-log plot is indicative of power-law scaling. Here power-law scaling is evident over times ranging about 10 and about 100 s. D) The ensemble-average of the power-spectrum 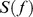 of the time series 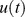. The dashed line with 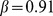 constitutes a linear least squares fit to the data (correlation coefficient, 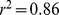).

A preliminary examination of the group 1 flight complete paths revealed that the distribution of loop lengths has an inverse power-law rather than an exponential one. The MLE are 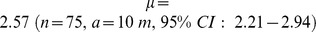 and 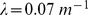. The Akaike weight for the maximum likelihood inverse power-law distribution was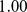, indicating that the inverse power-law distribution has stronger support than the alternative exponential distribution. When the loop-length distribution is assumed to be truncated at the length of the longest observed loop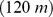, the MLE are 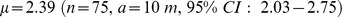 and 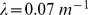, and the Akaike weight for the maximum likelihood inverse power-law distribution is again 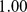. The null hypothesis that the observed data does indeed come from an inverse power-law distribution cannot be rejected 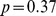. The close correspondence between the maximum likelihood inverse power-law and the data is illustrated in the plot of the survival function (Figure 4A). Our loop-length data are seen to exhibit inverse power-law scaling over more than one order of magnitude.

Our data for the first digit distribution is consistent with loop lengths coming from an inverse power-law distribution with 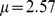
(Figure 4B). Outcomes from the time-series and spectral analysis provide further support for Lévy characteristics. Our time-series data are characterized by 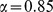 (linear least squares regression on log-log scales for times between 10 and 100 s, 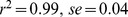) (Figure 4C) and our spectral data are seen to approximately follow a power-law scaling with 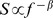 where 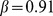
(linear least squares regression on log-log scales for frequencies between 1 and 100 s^−1^, 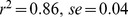) (Figure 4D). The result of time-series analysis 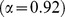 is indicative of Lévy flight characteristics with 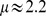 and is consistent with the result 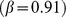
of the spectral analysis since 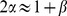.

### Evidence for Initiation of Foraging

There were gaps in some of the first flights of over 1 minute; and some of these were located over the patch of flowering field beans in the near vicinity of the colony (Figure 3A; Figure S3), but we cannot confirm the extent to which bumblebees start foraging on their first flights since no tracked bees returned from their first flights with pollen loads (although one bee that was not tracked did), although they may have sampled nectar.

On the second flights, there were gaps in the tracks which overlay forage areas and could indicate foraging at longer distances from the colony. One direct piece of evidence for foraging on a second flight was collected: Bee T348 (Figure S3) was actually observed foraging on hogweed (*Heracleum sphondylium L.)* located 30 m from the colony before returning to the colony. There were one or two flowering plants in this location next to a wooden Rothamsted moth light trap (1.5 m high; [38]) at the edge of a cereal field, and five tracks show evidence of the tracked bees spending time at this location.

The third flights have longer gaps (29–60 min) over areas of forage; and the experienced bees (group 3) show clear outward tracks to an area, followed by a long gap (14–108 min) interpreted as foraging (although possibly beyond radar view) and a defined return track to the colony; similar to the experienced foragers tracked in Osborne et al. [29]. These are goal-oriented vector flights and do not show characteristics of learning or exploring the landscape via loops or arcs. Of the eight experienced bees, there is direct evidence of foraging for four of them: three returned with pollen from plants that were located in the vicinity of the last radar signal of the outward track (lupin, poppy and field bean) whilst the fourth (T343) was seen on the hogweed plant near the moth trap. Almost all the experienced foraging flights went towards the south-west (Figure 3C). There were patches of lupins and field beans in this area, but there was also forage available in other directions – particularly in an area of gardens to the north-east. The wind direction during the study was predominantly from the south-west (Figure 3D) so all the tracked group 3 bees were flying upwind on their outward flight.

## Discussion

We have illustrated the ontogeny of bumblebee flights from naïve explorer to effective forager in a series of animated flight tracks (Figure S2), and shown the bees’ expanding use of space around the colony as they become more experienced (Figure 2A–C). The data were collected from bees in one colony in one location and, although this is often the case for such intricate behavioural studies of free flight [14], [17], [23], [27], inference should still be made with caution. Despite this limitation, it is the only dataset available to our knowledge to show the transition in flight between naïve exploration and foraging in bumblebees. The results indicate that bumblebees are impressively fast at learning the location of home, the location of food and memorizing efficient straight routes between these goals [2], [14], [39]. Analyses of simple parameters showed the bees few faster, further, straighter and covered less angular range around the colony as they became more experienced (Figure 3). It is striking that the bumblebees in this study made their first flights within a few days of emerging from pupae (average: 2 days), and started collecting pollen within a day or two of their first flight (also in [40]). One bumblebee in our study (not tracked) even brought back pollen on her first flight. This speed of development from naïve bee to forager is faster than documented for honeybees, who spend several days in a colony as nurse bees [38] before flying out of the colony, and perform many orientation flights before foraging effectively [27]. In future studies it would be interesting to compare whether the level of stores and demand in the colony alter the speed of progression to foraging.

### Learning, Exploring and Searching

How much time or effort is spent on learning and how much is spent on searching during these preliminary flights? Wei & Dyer [17] hypothesized for honeybees that duration of the ‘learning flight’ portion of a trip (meaning the small scale arcs in front of the colony) relates to investment in learning and stated that the bees fly off in one direction after the ‘learning phase’ suggesting they are then involved in another activity. Biesmeijer & Seeley [41] separate honeybee ‘orientation flights’ from the ‘search trips’ of scouts by using the duration of the excursion and the return of nectar and/or pollen to distinguish between them, which was pragmatic given that they did not have access to trajectory data. However the bumblebees’ complex and gradually expanding flight patterns illustrated here suggest that phases of orientation, learning and searching may not be easily separable, at least for bumblebees.

As expected, bumblebees demonstrated arcing behaviour at the beginning and end of their preliminary flights (1^st^ to 3^rd^ trips), turning to face the colony at distances of 0–2 m, to gain visio-spatial and olfactory information about the location of the colony and landmarks in the vicinity [6], [9], [16]. The radar tracks also show evidence of either arcing or fine scale looping around the colony entrance at distances of up to 10 m (Table 1; Table S1, Figure S2). Whilst the data are not at a high enough resolution to confirm their similarity to the very fine scale arcs seen by an observer, it is indicative of a learning phase at a larger scale than previously reported [9], [17].

The loops made by a bumblebee on her first flight gradually increase in size and are directed in different azimuthal directions. These first flights have Lévy characteristics that are consistent with the execution of optimal random looping searching strategy [25], adding to a growing body of evidence showing that several groups of animals use looping search patterns (e.g. honeybees, butterflies, moths and ants [20]–[23], [42], [43]. During these looping flights, the bees may not only be searching for the colony, but they may also be memorizing landmarks or scenes [44] to aid relocation of the colony; and they may also be searching for a food source. Our evidence, although limited to 38 tracks, suggests a high degree of multi-tasking within one or two flights (learning where home is, finding food and sampling it within one or two flights). How these neurologically complex tasks are prioritized or combined is an avenue of future study. An anecdotal example of this is the use of the small patch of hogweed, located 30 m from the colony by at least two bumblebees during preliminary flights. This patch of plants was not visible to the human eye from a long distance, although it was next to a wooden structure (the moth trap) so whilst the bees appeared to use the plants as a ‘service station’ to top up with nectar, the structure may also have been acting as a local landmark of known distance and bearing from the colony.

Our data suggest that bumblebees gather information on the landscape and forage sources from one or two complex flights covering all four quadrants around the colony, whereas Capaldi et al [27] showed that honeybees made a series simple single looped orientation flights, confined to a narrow sector around the hive. The two datasets are not directly comparable because they were collected in different years, with differing foraging availability, but the contrasts described raise a question of whether the simpler honeybee orientation flights could be shaped by their ability to use shared information on the location of forage resources [26], [45]. Have naïve honeybees already received information on where to find forage from dancing scouts in the colony? We find little evidence in the literature either for or against this hypothesis. Biesmeijer & Seeley [41] showed that 60% of ‘novice foragers’ engaged in their first few flights rely at least in part on acquiring information from following dances. But, the notion that differences in searching flight patterns may relate to differences in recruitment behaviour is supported by the fact that honeybees adopt optimal Lévy looping searching flights when they are triggered to search without the benefit of shared information [23], [24] after their hive centered navigational systems have been disrupted or rendered ineffective.

### Foraging

Bumblebees on their third flights showed lengthened and straightened flight paths which become, with further experience, straight vector flights to and from a forage location (similar to those illustrated in [29], [46]. Of particular interest in this study was that all of the experienced foragers were flying to the south west of the colony, where field beans were flowering. The landscape had patchy forage available in all directions from the colony (Figure S1). This strong bias in direction, with the bees flying in a predominantly upwind direction on their outward flights leads us to hypothesize that the floral olfactory cues may be providing strong directional information guiding their choice of forage over hundreds of metres. Bumblebees are known to share olfactory information in the colony when stimulating other bees to forage, even if they do not communicate location of that forage [26], [47], but in honeybees it is generally considered a short range cue if used directly [48], [49]. Examining the cues used by bumblebees to find forage sources was beyond the scope of this study, but further research to discover the scale over which bumblebees use visual and olfactory cues to make foraging decisions would help predict resource use. Seeley [50] suggests that if bees can utilize floral scent over hundreds of metres, then individual exploration is a very effective way of finding food in the landscape, without recruitment.

In summary, tracking bumblebees from their first flights to experienced foragers has shown their capability to quickly learn the location of home and the location of forage resources in a complex landscape, using a series of arcing and multiple looping flights (predicted by [45]), followed by vector flights. The general characteristics of these looping flights can be used to start to predict how bumblebees will find resources in complex landscapes. Further progress in elucidating how bees learn to utilize their landscape would be made by tracking individuals over sequential flights and simultaneously monitoring behaviour of individuals in the colony (sic [41]), together with repeated studies on different colonies with differing recruitment behaviour. These are required to tease apart the mechanisms of the apparent multi-tasking as the bees learn and explore the landscape.

## Supporting Information

Figure S1 **Diagrammatic map of the landscape used for tracking bumblebee flights.**
(PDF)

Figure S2 **Animated video sequences of the radar tracks of 28 bees tracked on complete flights.**
(ZIP)

Figure S3 **Plots of each tracked complete flight, overlaid on the landscape map, and illustrating gaps in radar signals of over 1 minute and over 3 minutes.**
(PDF)

Table S1 **Summary data for each tracked bumblebee flight used in the analyses.**
(PDF)
