# Supplementary material for: The Ontogeny of Bumblebee Flight Trajectories: From Naïve Explorers to Experienced Foragers
Source: PLoS One. 2013 Nov 12;8(11):e78681. doi: 10.1371/journal.pone.0078681 (PMC3827042; doi:10.1371/journal.pone.0078681)
Supplement: Figure S1 — Diagrammatic map of the landscape used for tracking bumblebee flights. (PDF) [file pone.0078681.s001.pdf]

Supporting Information: Figure S1: Diagrammatic map of the landscape used for tracking bumblebee flights. Temperature, wind speed and direction were recorded at 1.9 m above the ground at the five meteorological stations.

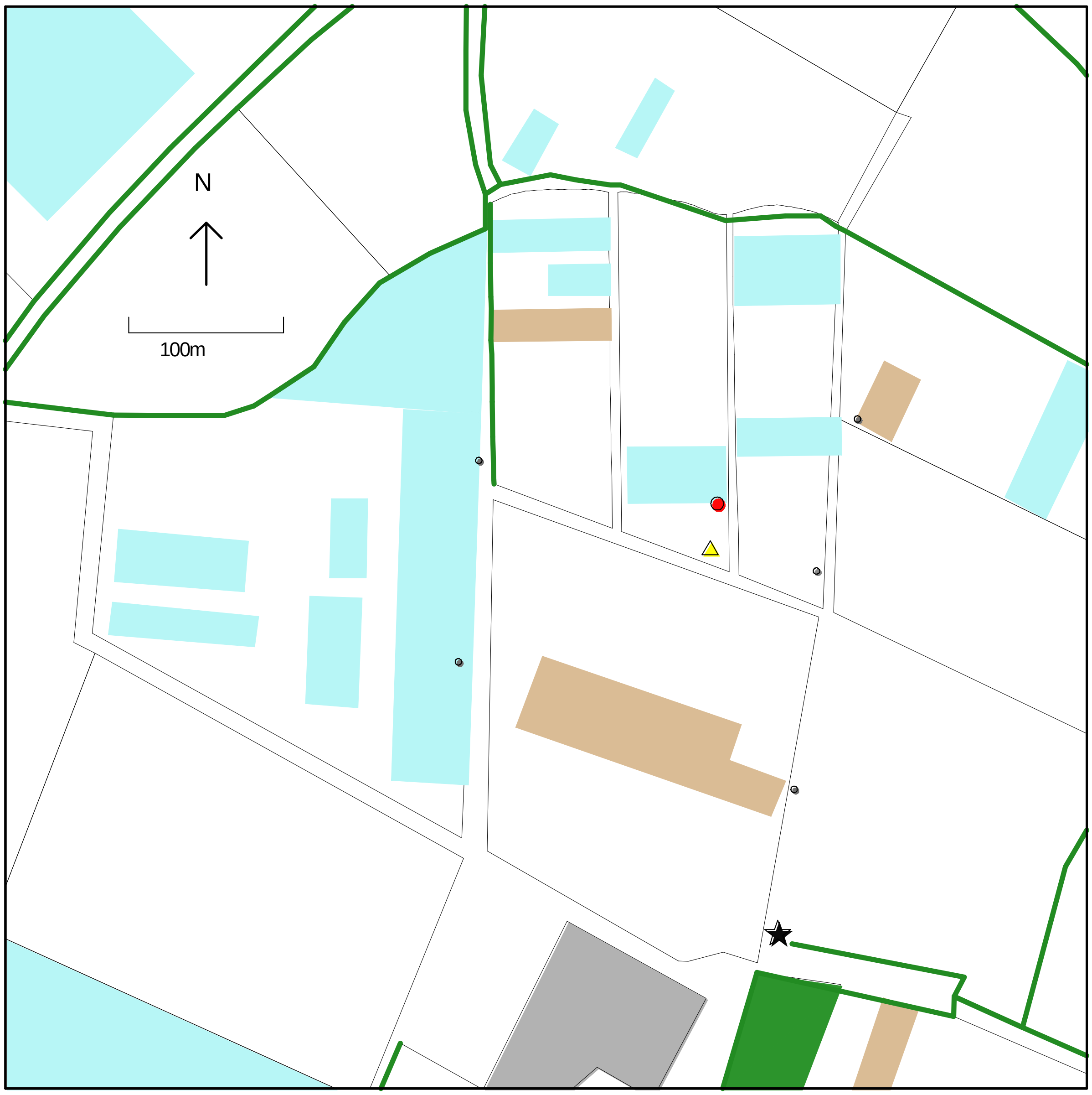

Key

- Field boundaries
- Hedgerows and wooded area
- Patches of crops or flowering plants providing nectar and pollen
- Tall maize crop could cause some obstruction of radar signals
- Buildings

- Colony location
- Radar location
- 5 meteorological stations
- Location of moth light trap and hogweed plants

Supporting Information for:

PLoS ONE

The Ontogeny of Bumblebee Flight Trajectories: From Naïve Explorers to Experienced Foragers

Juliet L. Osborne,*[1,2] Alan Smith,[2] Suzanne J. Clark,[2] Don R. Reynolds,[3] Mandy C. Barron,[4] Ka S. Lim[2], Andy M. Reynolds[2]

[1] Environment & Sustainability Institute, University of Exeter, Penryn Campus, Penryn, Cornwall, TR10 9EZ, UK

[2] Rothamsted Research, Harpenden, Hertfordshire, AL5 2JQ, UK

[3] Natural Resources Institute, University of Greenwich, Chatham, Kent, ME4 4TB, UK

[4] Landcare Research, PO Box 40, Lincoln, 7640, New Zealand

*Corresponding author: J.L.Osborne@exeter.ac.uk
