# Supplementary material for: The Ontogeny of Bumblebee Flight Trajectories: From Naïve Explorers to Experienced Foragers
Source: PLoS One. 2013 Nov 12;8(11):e78681. doi: 10.1371/journal.pone.0078681 (PMC3827042; doi:10.1371/journal.pone.0078681)
Supplement: Figure S2 — Animated video sequences of the radar tracks of 28 bees tracked on complete flights. (ZIP) [file pone.0078681.s002.zip › Fig S2 legend Osborne 2013.pdf]

Supporting Information: Figure S2 legend: Animated radar tracks of 28 bees tracked on complete flights, to show sequence and direction of flights, and to illustrate arcing and looping behaviour. Flights start at the nest; red dot indicates location of signals indicating bee's position; grid indicates scale of 10 m. Sequences are not in real time (actual time frame when tracking is 3 seconds between radar signals) and gaps longer than 3 seconds are not indicated. See maps in Figure S3 to show where there were gaps in tracks. Files are named according to the track numbers in Table S1 and Figure S3.
