# Supplementary material for: The Ontogeny of Bumblebee Flight Trajectories: From Naïve Explorers to Experienced Foragers
Source: PLoS One. 2013 Nov 12;8(11):e78681. doi: 10.1371/journal.pone.0078681 (PMC3827042; doi:10.1371/journal.pone.0078681)
Supplement: Figure S3 — Plots of each tracked complete flight, overlaid on the landscape map, and illustrating gaps in radar signals of over 1 minute and over 3 minutes. (PDF) [file pone.0078681.s003.pdf]

Supporting Information: Figure S3: Plots of each tracked complete flight overlaid on the landscape map (see key in Figure S1). The maps are presented at two scales depending on the range of the track. Legend under each plot shows track number, the experience of the bee (trip number) and the number and length of gaps longer than 3 minutes; together with evidence of foraging. Orange ovoids have been super-imposed on the tracks to show where there were gaps between signals over 3 minutes and evidence of foraging (indirect or direct) is noted in the legend.

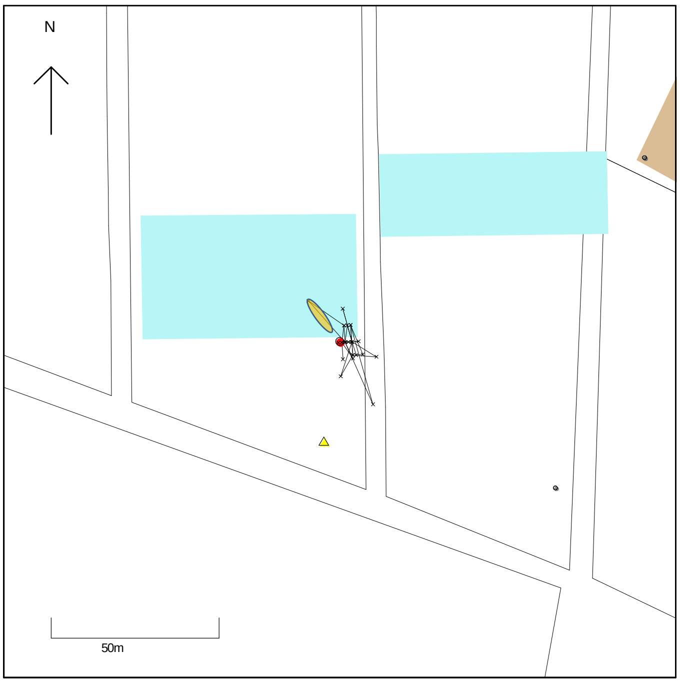

T302: 1st trip; Gaps (184s - over forage)

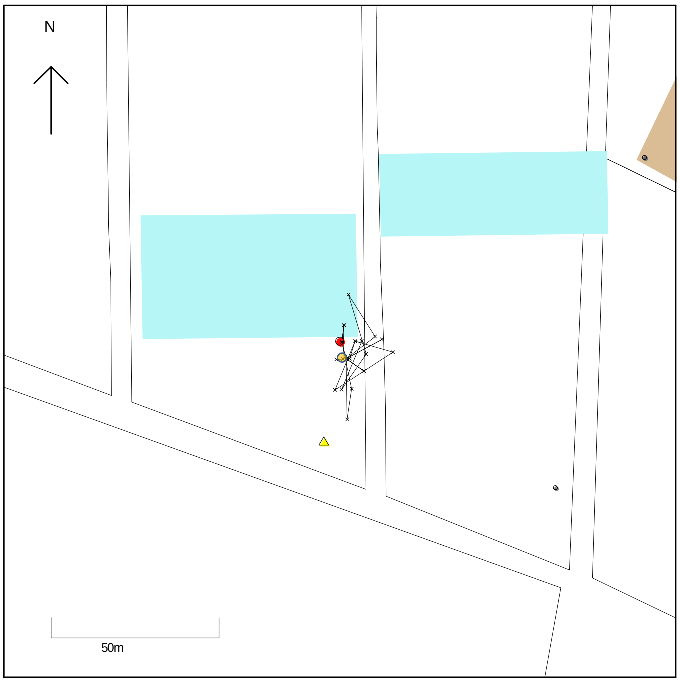

T305: 1st trip; Gaps (405s)

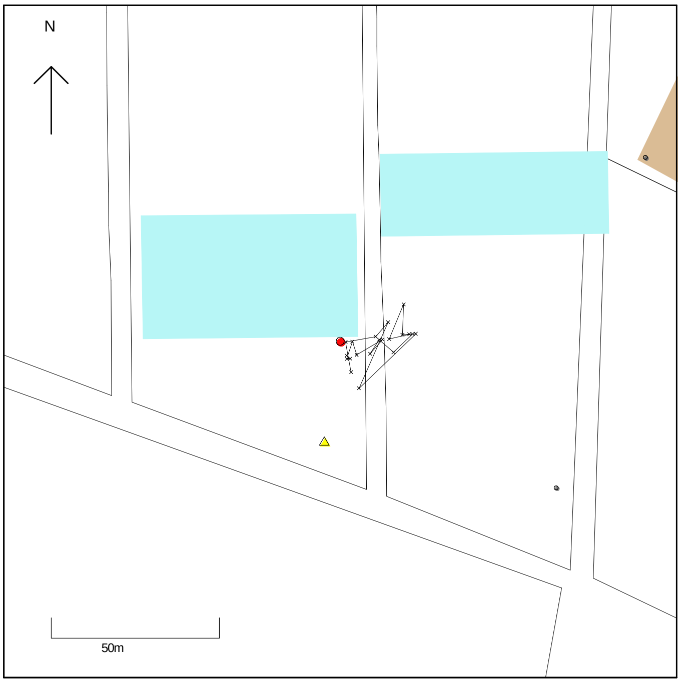

T311: 1st trip; Gaps (none)

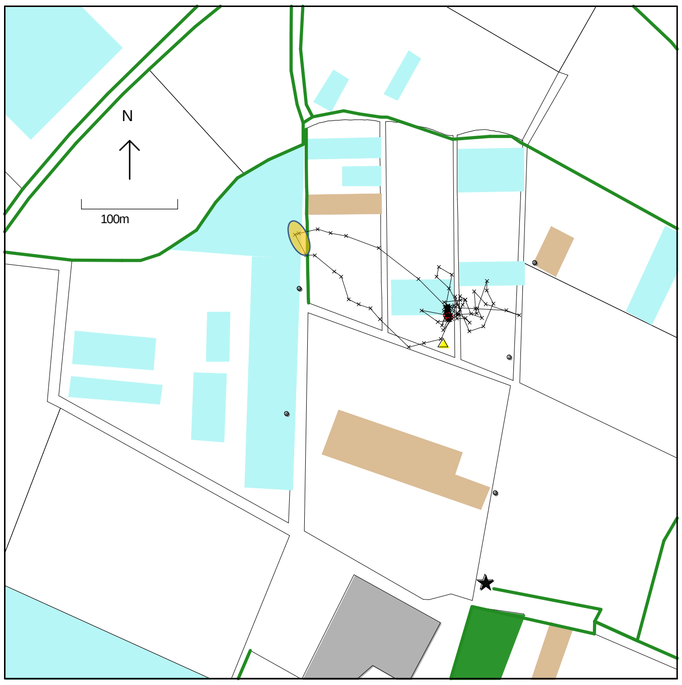

T314: 1st trip; Gaps (1110s+1177s - over hedge near forage)

Group 1 bees (continued)

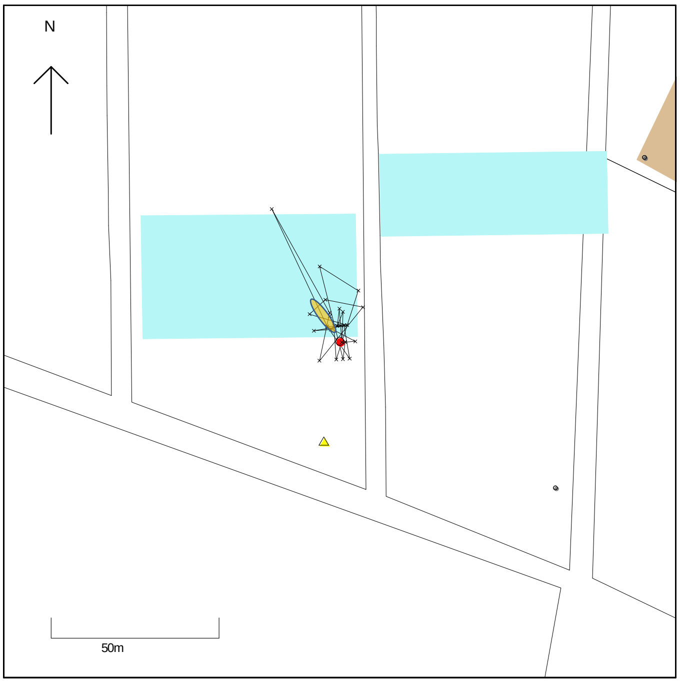

T315: 1st trip; Gaps (none)

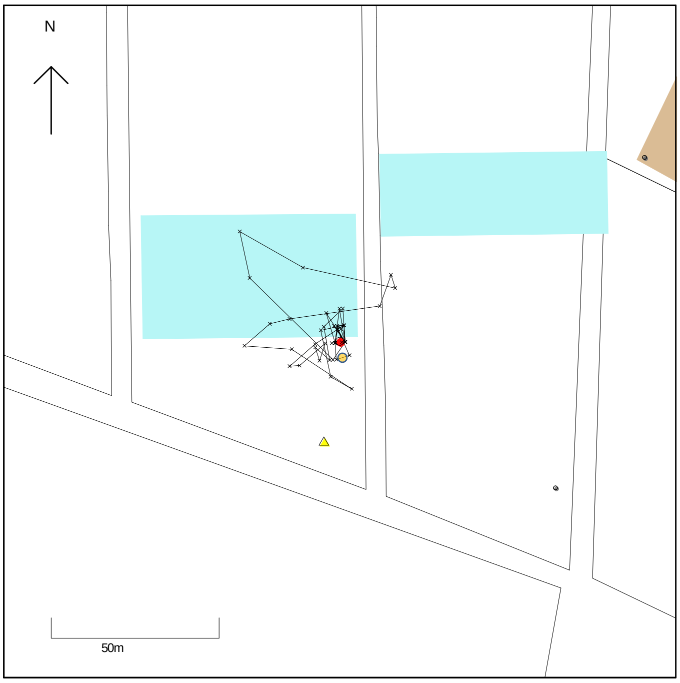

T318: 1st trip; Gaps (none)

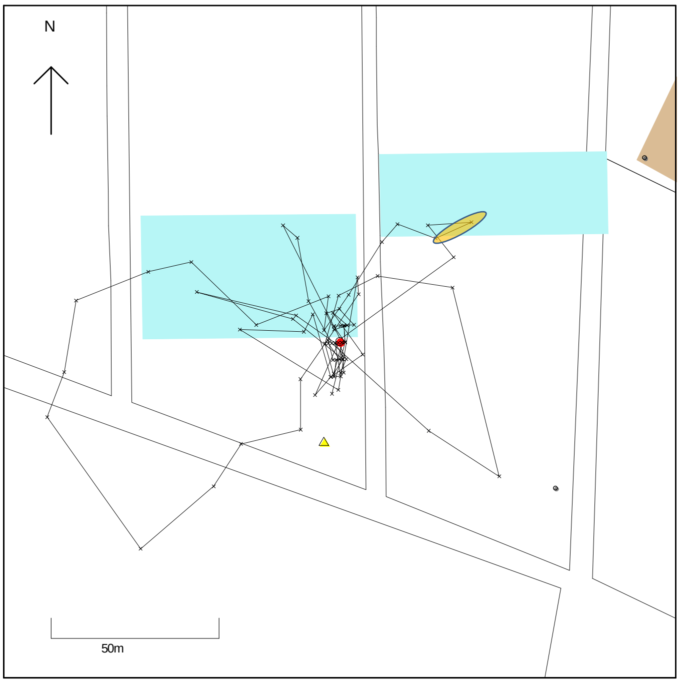

T319: 1st trip; Gaps (190s over forage)

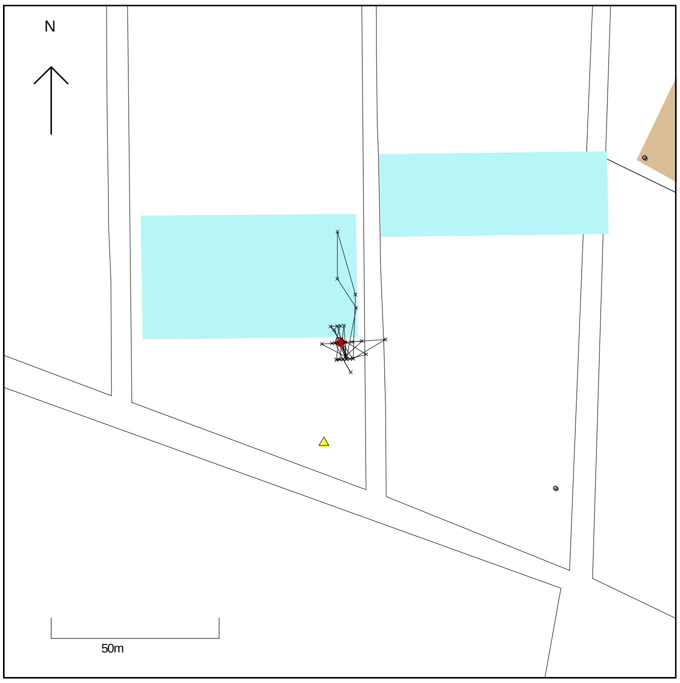

T320: 1st trip; Gaps (none)

Group 1 bees (continued)

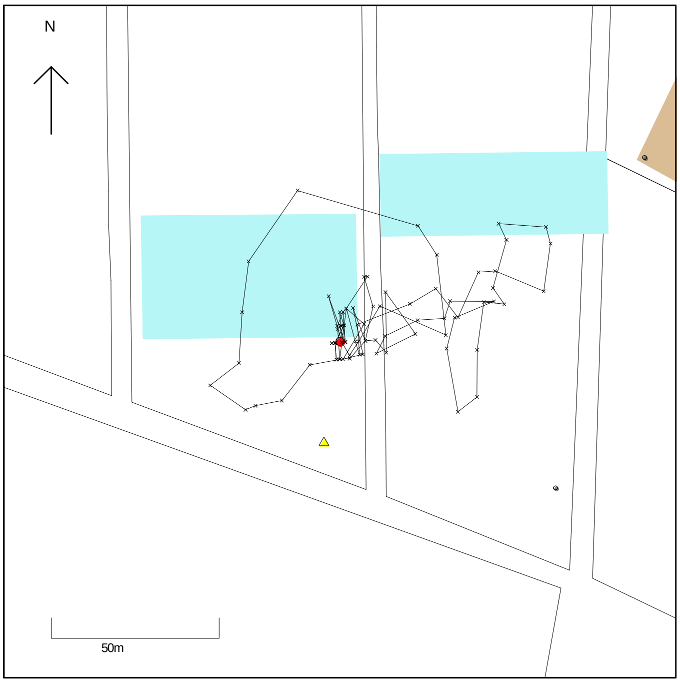

T325: 1st trip; Gaps (none)

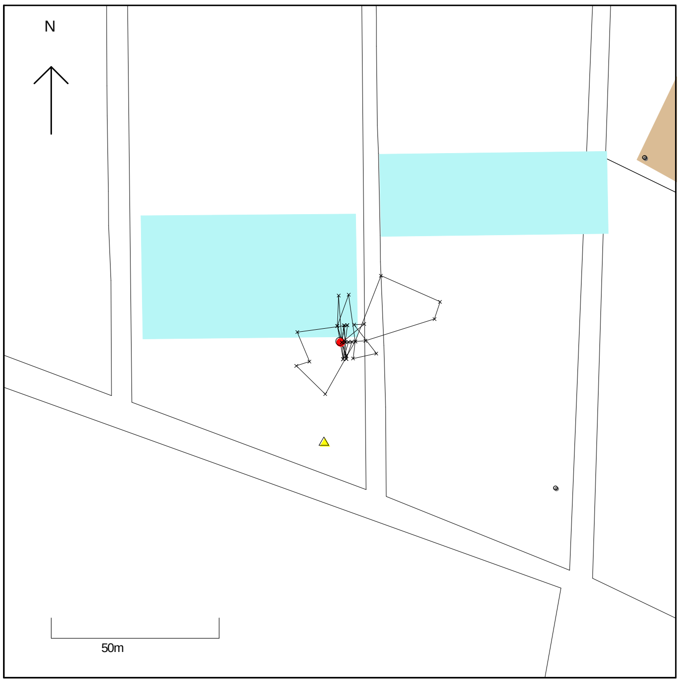

T330: 1st trip; Gaps (none)

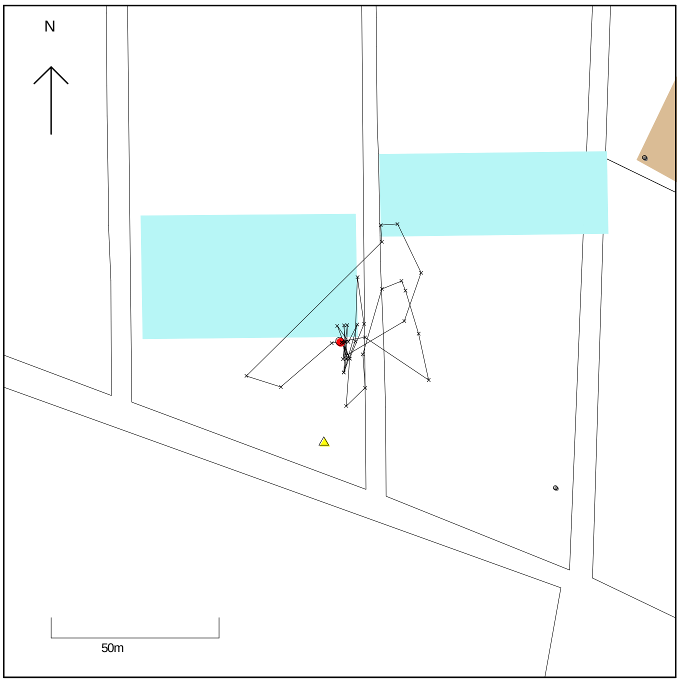

T339: 1st trip; Gaps (none)

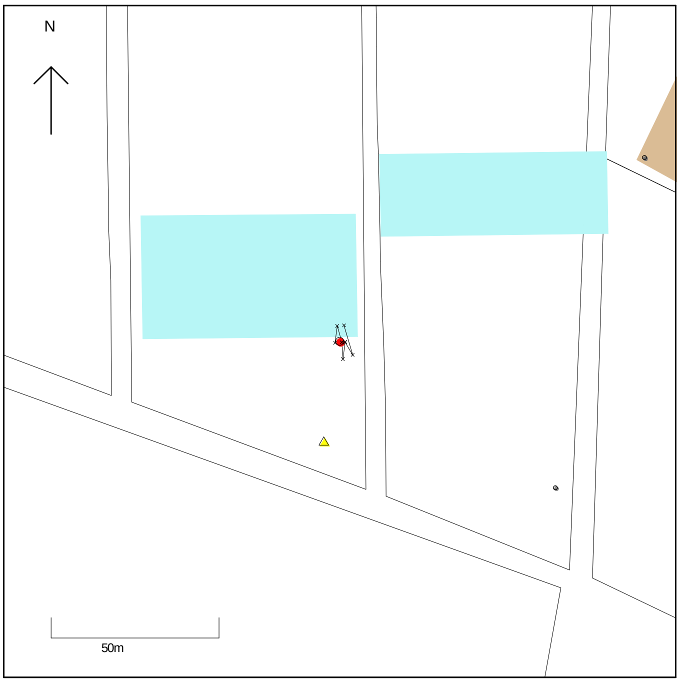

T340: 1st trip; Gaps (none)

Group 1 bees (continued)

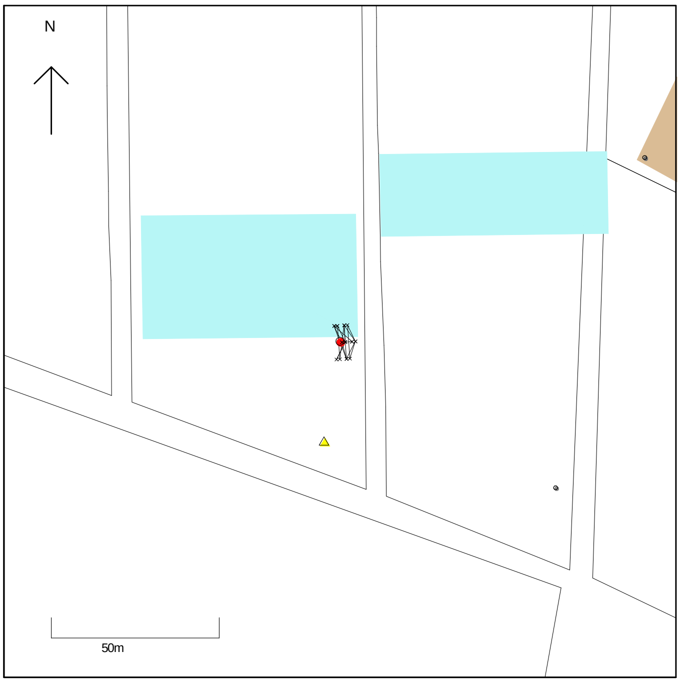

T341: 1st trip; Gaps (none)

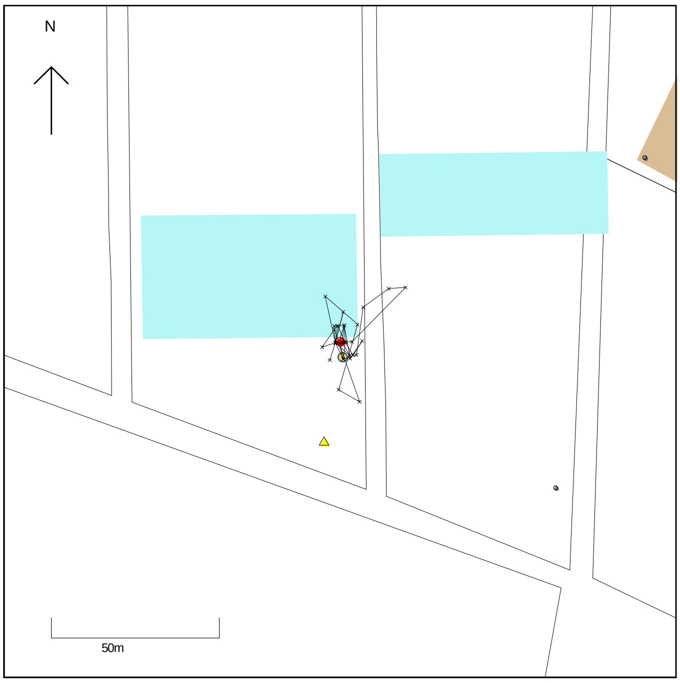

T341ex: 1st trip; Gaps (none)

Group 2 bees (2nd trips)

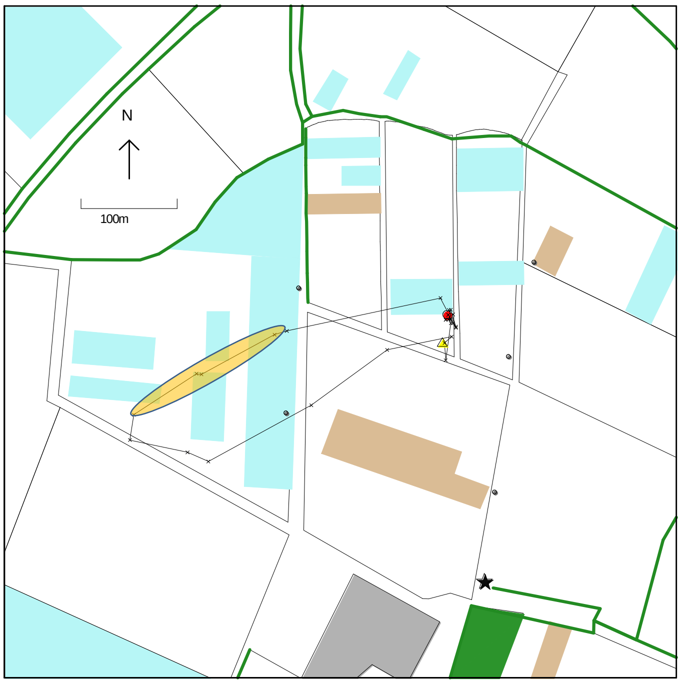

T304: 2nd trip; Gaps (85s + 417s +114s over forage)

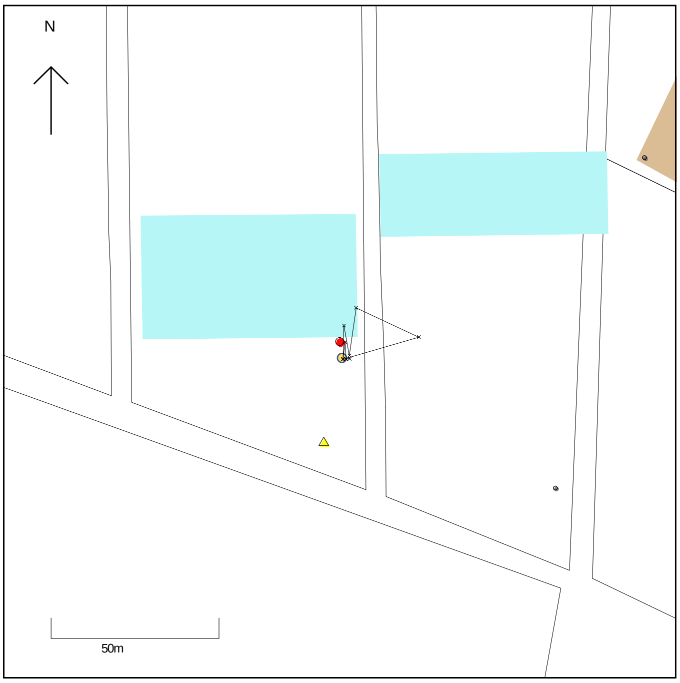

T310: 2nd trip; Gaps (none)

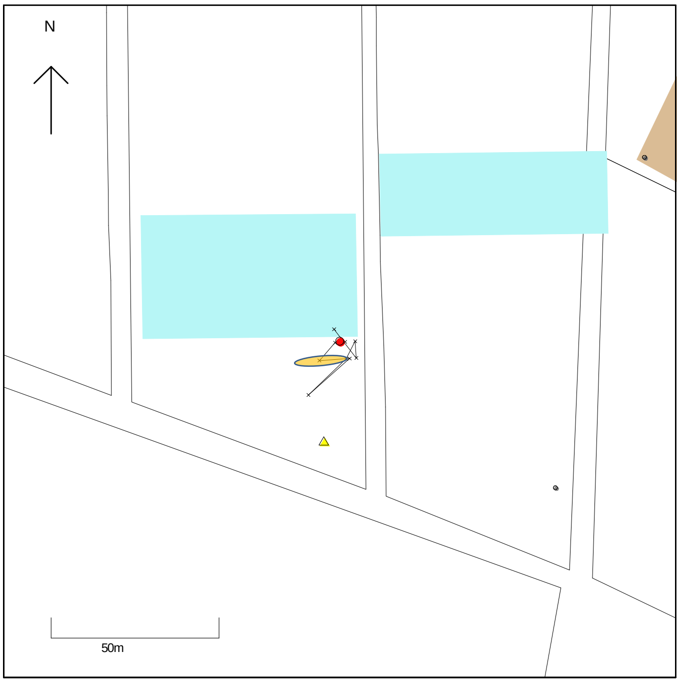

T336: 2nd trip; Gaps (183s)

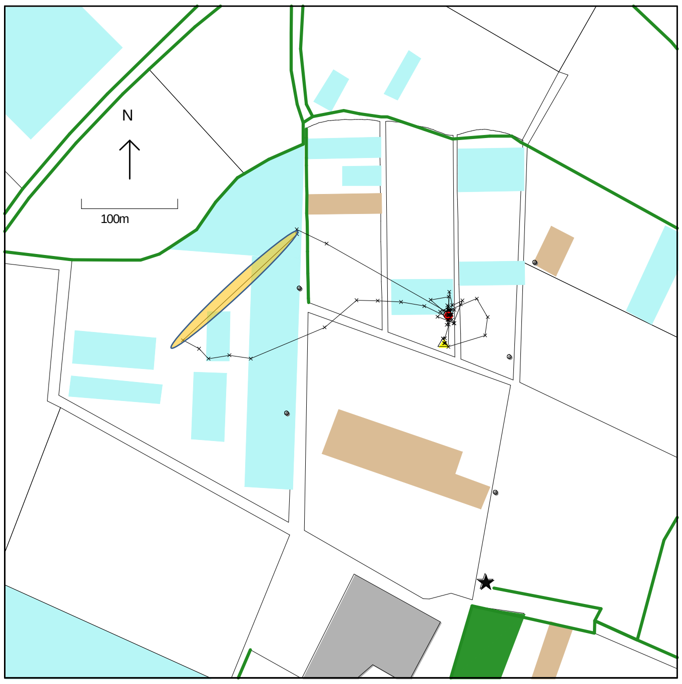

T348: 2nd trip; Gaps (629s over forage); observed foraging on hogweed (at yellow triangle)

Group 2 bees (3rd trips)

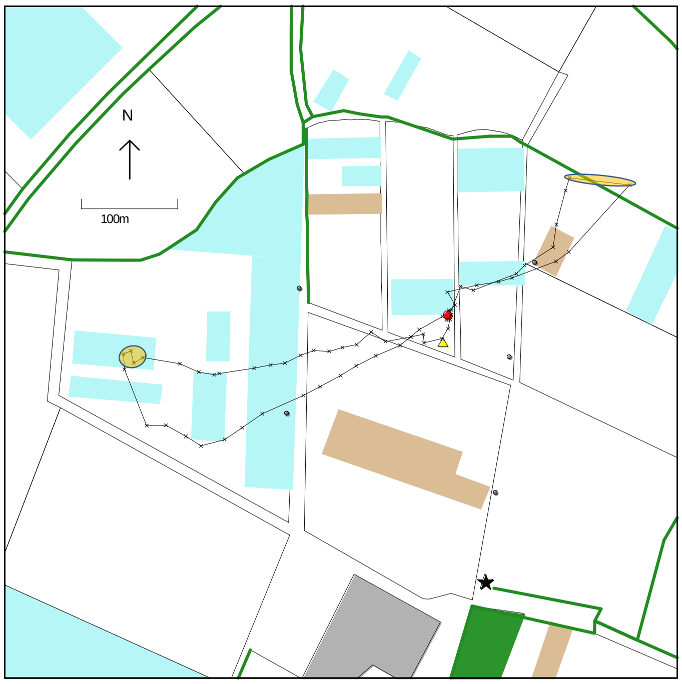

T317: 3rd trip; Gaps (1041s over hedge; 500s + 3227s over forage)

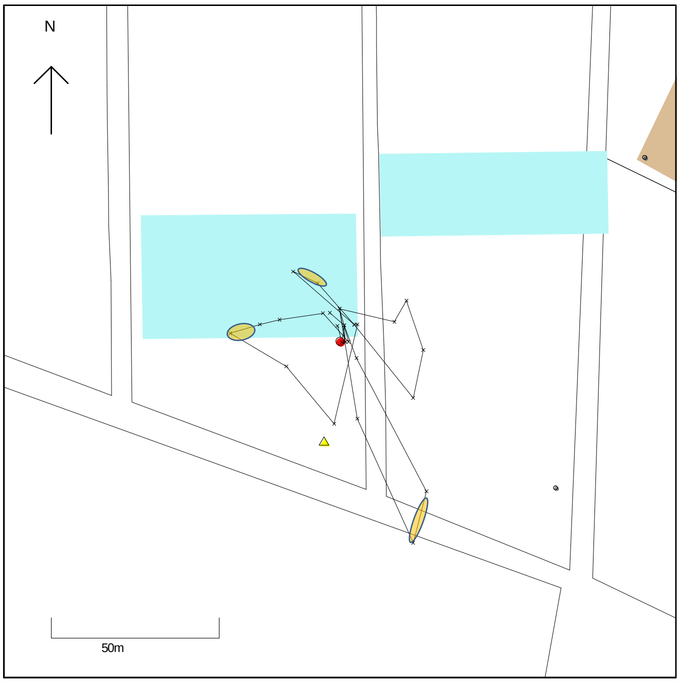

T335: 3rd trip; Gaps (403s, 1796s + 1005s over forage)

Group 3 bees

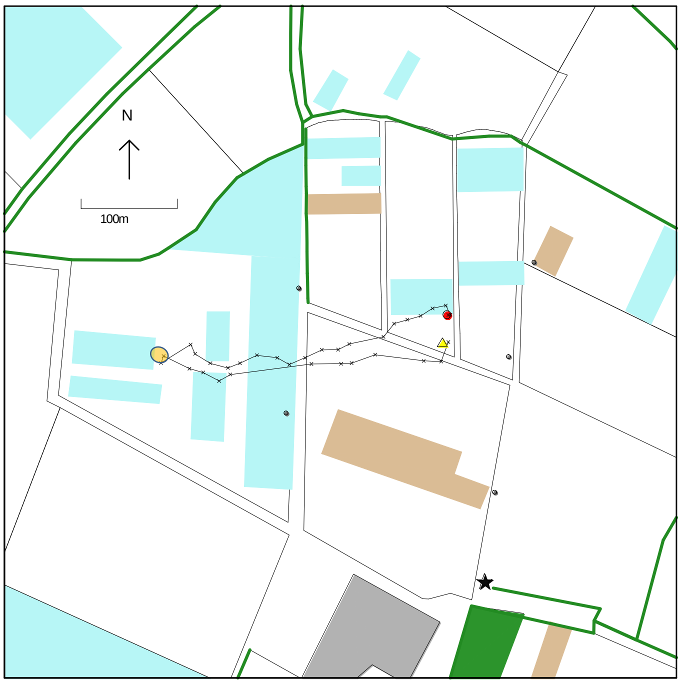

T301/303: Experienced >6th trip; Gaps (876s next to lupin patch); returned with LUPIN pollen

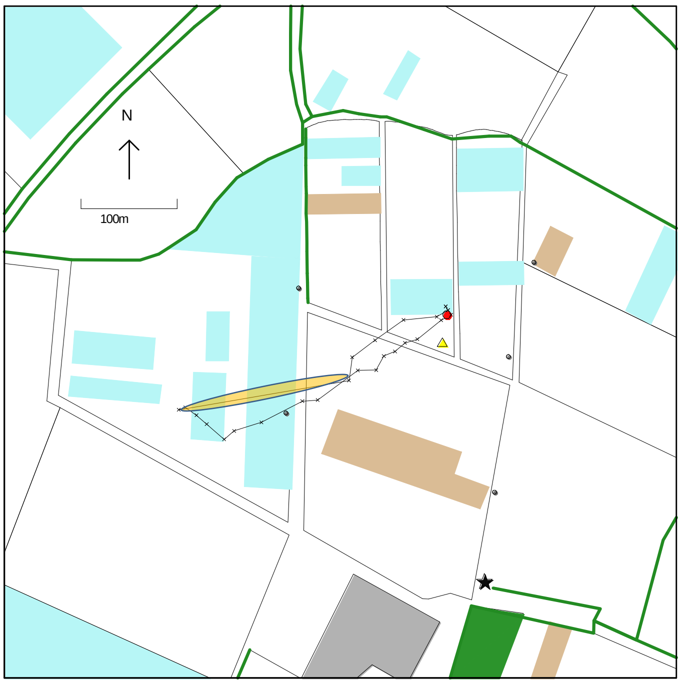

T322: Experienced >6th trip; Gaps (6471s over forage)

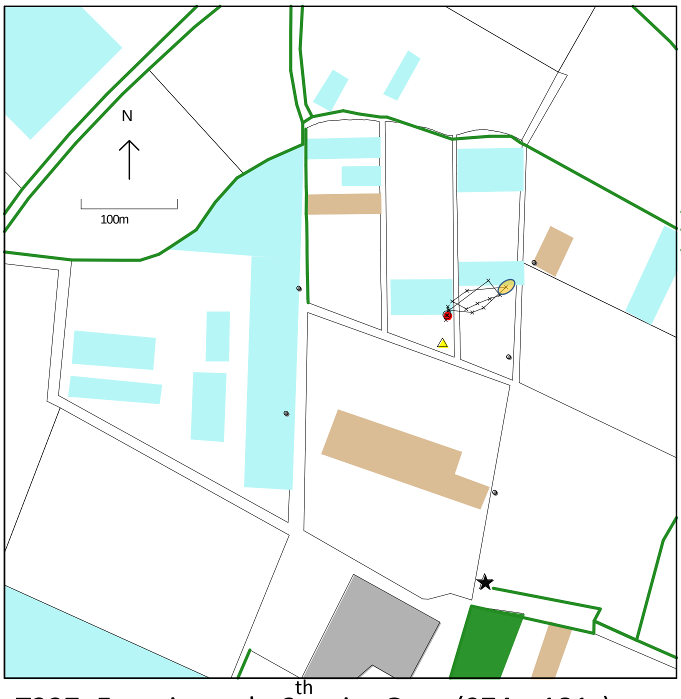

T327: Experienced >6th trip; Gaps (974s, 181s)

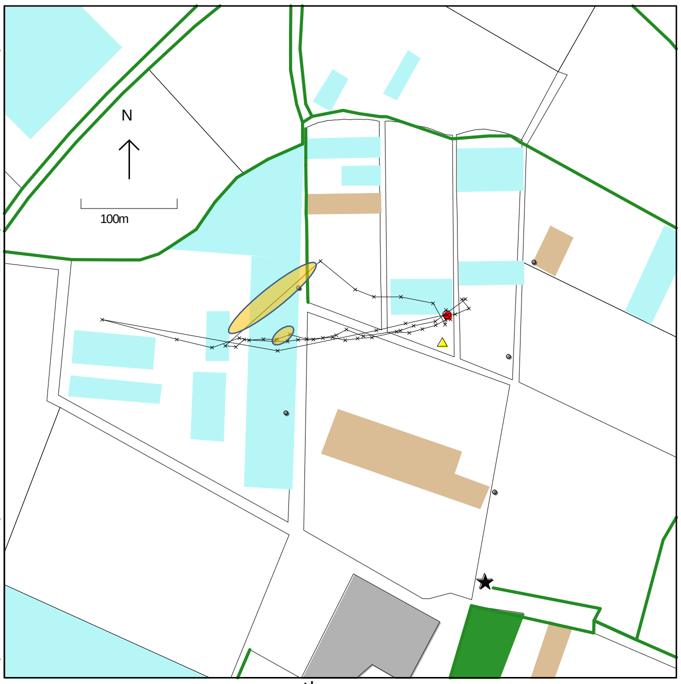

T338: Experienced >6th trip; Gaps (4392s + 2719s both over grass with poppies), returned with POPPY pollen

Group 3 bees (continued)

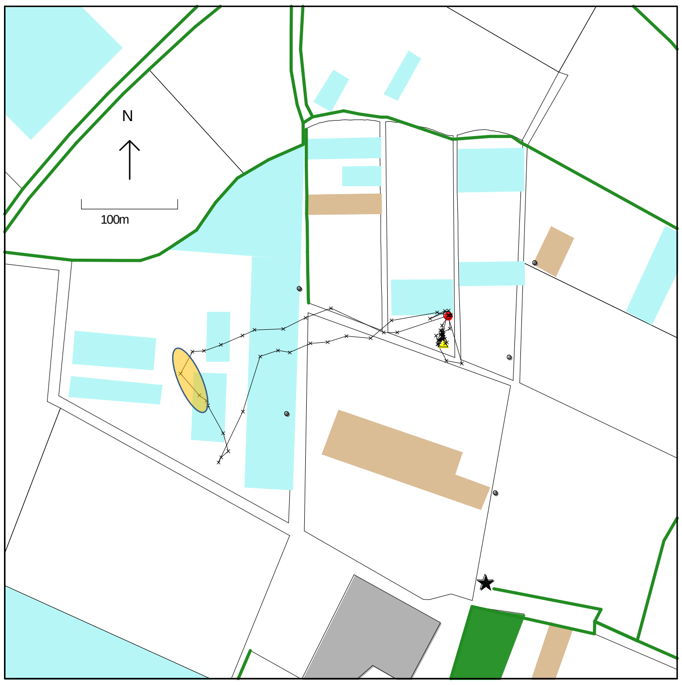

T343: Experienced >6th trip; Gaps (102s+64s+93s, 202s); observed foraging on hogweed (at yellow triangle)

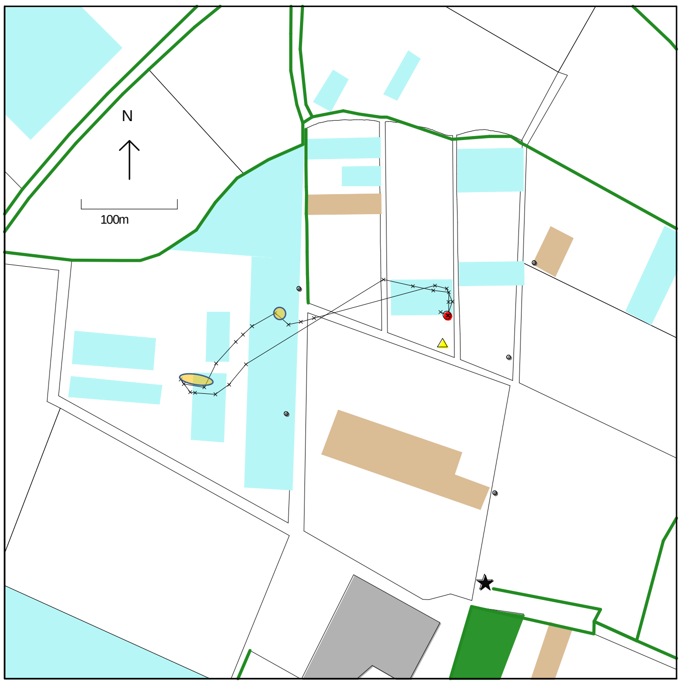

T344: Experienced >6th trip; Gaps (84s+66s+73s over forage)

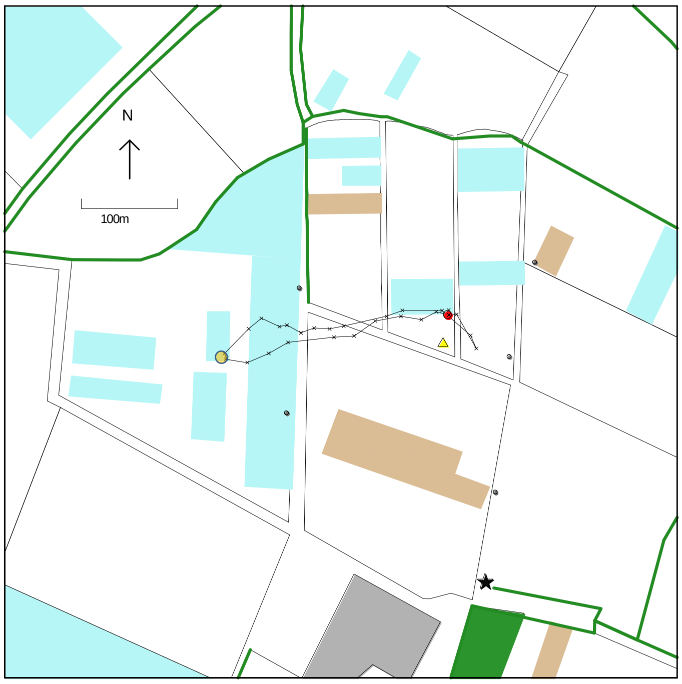

T345: Experienced >6th trip; Gaps (3002s over forage)

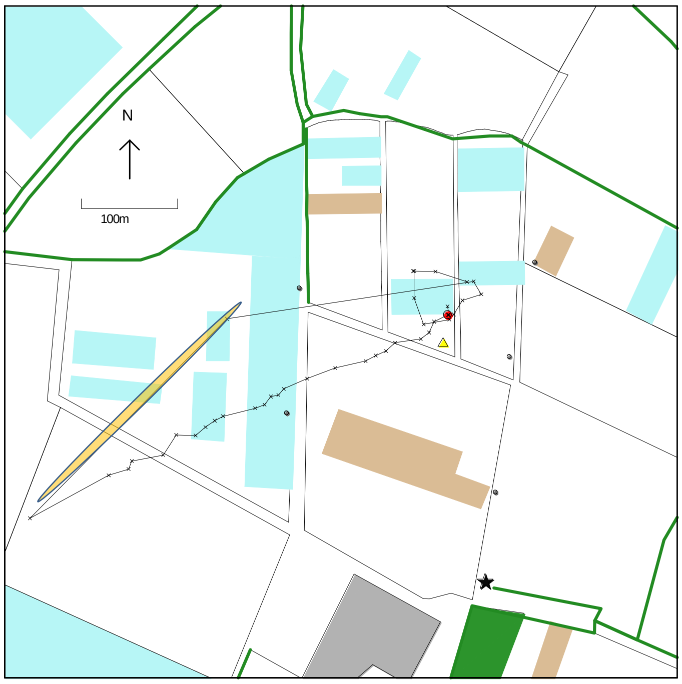

T346: Experienced >6th trip; Gaps (4337s on return); returned with FIELD BEAN pollen

Supporting Information for:

PLoS ONE

The Ontogeny of Bumblebee Flight Trajectories: From Naïve Explorers to Experienced Foragers

Juliet L. Osborne,* [1,2] Alan Smith,[2] Suzanne J. Clark,[2] Don R. Reynolds,[3] Mandy C. Barron,[4] Ka S. Lim[2], Andy M. Reynolds[2]

[1] Environment & Sustainability Institute, University of Exeter, Penryn Campus, Penryn, Cornwall, TR10 9EZ, UK

[2] Rothamsted Research, Harpenden, Hertfordshire, AL5 2JQ, UK

[3] Natural Resources Institute, University of Greenwich, Chatham, Kent, ME4 4TB, UK

[4] Landcare Research, PO Box 40, Lincoln, 7640, New Zealand

*Corresponding author: J.L.Osborne@exeter.ac.uk
