# Supplementary material for: The Ontogeny of Bumblebee Flight Trajectories: From Naïve Explorers to Experienced Foragers
Source: PLoS One. 2013 Nov 12;8(11):e78681. doi: 10.1371/journal.pone.0078681 (PMC3827042; doi:10.1371/journal.pone.0078681)
Supplement: Table S1 — Summary data for each tracked bumblebee flight used in the analyses. (PDF) [file pone.0078681.s004.pdf]

Supporting Information: Table S1: Summary data for each tracked bumblebee flight. Group refers to experience as used in statistical analyses. Grey font indicates tracks that were not complete (part) because the bee was not radar-tracked returning home – in these cases, data were only used for average ground speed, quadrant, arc and loop analyses (not distance parameters). # CHP = Convex Hull Polygon, **$** IQR = Interquartile range of bearings, **†** Evidence of arcs or Turn Back & Look flights (TBL) at the start and end of the flights were recorded visually within 2m of colony entrance and on the radar track within 10m of colony entrance.

Bee	Track no	Age when tracked (days)	Trip no.	Group	No. fixes in track	Max displace-ment (m)	Total distance (m)	Duration (s)	CHP# area (m^2)	IQR$ (deg)	No. quad-rants	Mean (±s.e.) step speed for gaps < 15s	no. of gaps < 15s	no. gaps ≥ 15s	no. gaps 1-3 mins	no. gaps > 3 mins	†Visual: TBL/arcs @ start	†Radar: arcs @ start	†Radar: arcs @ end	Approx no. loops	Foraging evidence
Y39	302a-b	6	1	1	31	20.9	213.1	520	280	65.2	3	1.72 ± 0.3	26	4	1	1	Y	Y	Y	5	
Y59	305	3	1	1	30	23	242.6	875	332.1	70.2	3	1.39 ± 0.3	22	7	2	1	Y	Y	?	3	
Y72	311	2.5	1	1	21	22.8	145	123	268.1	42.7	2	1.53 ± 0.3	19	1	0	0	Y	N	N	1	
Y50	314	7	1	1	124	179.4	1510.1	3360	13807	94.3	4	2.68 ± 0.2	112	11	5	1	Y	Y	Y	5	
Y56	315	6	1	1	29	44.5	307.4	304	521.5	83.0	4	2.04 ± 0.3	25	3	1	0	N	Y	N	4	
W2	318	0.5	1	1	52	44.3	416.3	211	1428	109.8	4	2.19 ± 0.3	51	0	0	0	Y	Y	Y	3	
G42	319	10	1	1	84	89.6	1083.8	890	9964	176.7	4	3.23 ± 0.3	76	7	3	1	Y	Y	Y	6	
R28	320	2	1	1	42	33	287.6	184	401	153.0	4	1.96 ± 0.2	40	1	0	0	Y	Y	Y	3	
Y66	325	7	1	1	91	70.5	777.9	572	5081	92.6	4	2.2 ± 0.2	85	5	0	0	Y	Y	Y	6	
R30	330	<1	1	1	38	32.4	314.4	372	816	102.0	4	2.28 ± 0.4	33	4	2	0	Y	Y	Y	4	
W37	339	2	1	1	46	39.3	434.2	172	1683	84.1	4	2.76 ± 0.3	44	0	0	0	Y	Y	N	3	
W33	340	3	1	1	9	5.5	34	138	32	136.1	2	1.09 ± 0.4	5	3	0	0	Y	?	?	1	
W38	341	2	1	1	22	5.7	117.7	156	50	81.2	4	1.6 ± 0.3	17	4	0	0	Y	Y	Y	0	
R36	341ex	<1	1	1	46	25.6	268.2	340	513.5	149.0	4	1.23 ± 0.2	41	4	1	0	Y	Y	Y	4	
G53	313	9	1 (part)	1	22	*	*	*	*	*	4	2.57 ± 0.5	18	3	0	0	Y	Y	*	2	
W8	316	<1	1 (part)	1	29	*	*	*	*	*	2	0.97 ± 0.2	21	3	1	0	Y	Y	*	2	
Y78	323	3.5	1 (part)	1	54	*	*	*	*	*	3	2.49 ± 0.4	47	6	1	3	Y	Y	*	2	
W39	347a-d	4	1 (part)	1	127	*	*	*	*	*	4	3.00 ± 0.2	99	25	4	7	Y	Y	*	5	
Y47	304	5	2	2	29	354.7	946.7	776	27344	129.7	4	3.92 ± 0.8	19	9	2	1	Y	N	Y	3	
Y67	310	3.5	2	2	11	23.8	91.7	208	189	100.1	2	2.19 ± 0.9	9	1	1	0	N	Y	?	1	
R3	317	<1	3	2	60	340.8	1371.7	5074	53431	195.1	2	5.74 ± 0.3	54	5	1	3	N	N	N	2	
W19	335a-b	3	3	2	29	63.5	429.1	3531	2546	110.6	4	3.87 ± 0.6	22	6	2	3	N	N	Y	4	
W21	336	3	2	2	9	18.1	72.7	315	119.5	132.7	3	1.8 ± 0.3	6	2	1	1	Y	N	N	1	
W35	348a-b	5	2	2	61	276.3	1162.5	1228	25871	186.3	4	3.32 ± 0.4	49	11	1	1	Y	Y	?	3	hogweed foraging
Y58	312ab	5	2 (part)	2	18	*	*	*	*	*	4	2.85 ± 0.7	17	0	0	0	Y	Y	*	3	
W7	329	3	2 (part)	2	11	*	*	*	*	*	2	1.61 ± 0.5	8	2	0	1	Y	Y	*	1	
W12	326	2.5	3 (part)	2	14	*	*	*	*	*	1	3.63 ± 0.4	12	1	0	0	N	N	*	0	
Y12	301+3	6	7	3	33	301.6	674	1095	13570	15.3	1	5.55 ± 0.4	28	4	0	1	Y	N	N	1	lupin pollen
G47	322	11	~ 18	3	26	295.9	667.4	6703	10639	14.5	1	5.15 ± 0.7	21	4	1	1	N	N	N	1	
W3	327a-b	3	~ 22	3	16	68.5	278.1	1380	1220	54.6	1	3.96 ± 0.7	12	3	1	2	N	N	N	2	
Y1	338a-c	15	~ 66	3	50	358.8	1355.6	7380	20466	11.2	3	4.83 ± 0.4	44	5	0	2	N	N	N	3	poppy pollen
W38	343a-b	3	10	3	71	284.2	1102	1587	27840	68.9	2	3.3 ± 0.4	48	22	8	1	N	N	N	2	hogweed foraging
Y17	344	16	~ 81	3	28	285.1	714.5	424	14370	79.1	2	4.16 ± 0.5	20	7	3	0	N	N	N	1	
W37	345a-b	3	~ 16	3	29	235.4	610.2	3237	10447	21.4	2	5.22 ± 0.1	23	5	0	1	N	N	N	1	
Y20	346a-b	17	~ 125	3	40	483.4	1268.4	4673	33136	78.8	3	3.51 ± 0.3	32	7	0	1	N	N	N	2	field bean pollen
Y46	309	6	~10 (part)	3	26	*	*	*	*	*	1	4.06 ± 0.3	22	3	2	1	N	N	*	0	
Y59	321	7	~43 (part)	3	32	*	*	*	*	*	1	3.97 ± 0.2	30	1	0	0	N	N	*	0	
Y61	328	9	~97 (part)	3	8	*	*	*	*	*	1	7.11 ± 0.2	4	3	0	1	N	N	*	0	

Supporting Information for:

PLoS ONE

The Ontogeny of Bumblebee Flight Trajectories: From Naïve Explorers to Experienced Foragers

Juliet L. Osborne,* [1,2] Alan Smith,[2] Suzanne J. Clark,[2] Don R. Reynolds,[3] Mandy C. Barron,[4] Ka S. Lim[2], Andy M. Reynolds[2]

[1] Environment & Sustainability Institute, University of Exeter, Penryn Campus, Penryn, Cornwall, TR10 9EZ, UK

[2] Rothamsted Research, Harpenden, Hertfordshire, AL5 2JQ, UK

[3] Natural Resources Institute, University of Greenwich, Chatham, Kent, ME4 4TB, UK

[4] Landcare Research, PO Box 40, Lincoln, 7640, New Zealand

*Corresponding author: J.L.Osborne@exeter.ac.uk
